# Understanding Solvent-Induced Glass Transition in Polymer Thin Films Using Absorption–Desorption Isotherms

**DOI:** 10.1021/acs.macromol.5c02242

**Published:** 2026-03-02

**Authors:** Nayanathara Hendeniya, Sharif Tasnim Mahmud, Shaghayegh Abtahi, Boyce S Chang

**Affiliations:** † Department of Materials Science and Engineering, 1177 Iowa State University, Ames, Iowa 50011, United States; ‡ Micro-Electronics Research Center, Iowa State University, Ames, Iowa 50011, United States

## Abstract

The fundamental thermodynamic and mechanical underpinnings of polymer thin films exposed to solvent vapor are critical for the development of advanced nanolithography and high-performance coatings. This work investigates the solvent–polymer interactions of glassy thin films by using the solvent absorption–desorption isotherms. An analogous relationship to the Flory–Fox equation was observed between solvent–induced glass transition, swelling, Flory–Huggins interaction parameter, and molecular weight. Isothermal swelling measurements revealed that the glass transition trends are more robust in the absorption curve compared to desorption, contrary to previous reports. Excess osmotic pressure analysis of the isotherm provides a measure of the degree of physical aging in thin films annealed below the glass transition. This is further validated in the ordering of block copolymer (BCP) films annealed at low solvent activity. In agreement with the thermal analysis, free-surface plasticization effects become the most prominent below 100 nm. However, solvent annealing is largely dependent on solvent mass transport, as made evident by the strong dependence on solvent viscosity. From these observations, four general types of isotherms are identified that graphically capture distinct solvent–polymer interaction regimes. More broadly, these results inform solvent vapor annealing-induced self-assembly, sequential infiltration synthesis, membrane-based separations, adsorptive processes, and swelling-based responsive materials design.

## Graphical abstract

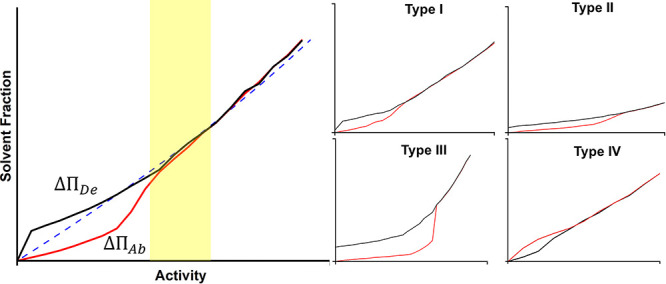

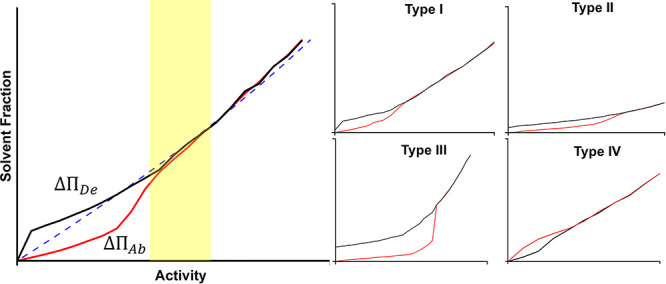

## Introduction

Understanding the unique properties of polymer thin films, arising from nanoscale confinement, is critical across a range of technological fields, from advanced nanolithography to high-performance coatings.^1–3^ Thin film preparation methods using solvents, such as solution casting and spin coating, allow precise control over film thickness, enabling uniform film deposition.^4^ Here, the solvent evaporation rate determines the final morphology and properties of the film.^5–7^ Spray coating methods are ideal for large-scale production, with no limitation on substrate size and less material wastage.^8^ Furthermore, thermally sensitive materials such as precured inks for 3D printing,^9^ polypeptides,^10,11^ polynucleic acids,^12^ and polymer complexes^13,14^ are exclusively solvent processed. In addition, solvent vapor annealing (SVA) offers a unique opportunity to exploit swelling-induced self-assembly of block copolymers (BCP), which mitigates the kinetic barriers associated with high χ systems.^15,16^ Solvent selectivity plays a key role in the morphological evolution and orientation control in the SVA of BCPs.^14,17,18^ Hence, controlling the solvent environment (concentration, temperature, vapor pressure, etc.) provides a powerful toolbox for kinetically engineering nanostructures.^15,19^

In addition to material deposition, understanding polymer–solvent interactions is crucial for separation membranes, where solvent mediation is deliberately devised to ensure selective transport. In bioprinting, solvent interactions affect flow properties, curing mechanisms, and layer adhesion—vital for creating functional parts with consistent properties.^20^ Finally, for controlled release systems, polymer swelling in physiological fluids is utilized as a minimally invasive delivery of cells or drugs.^21^ Therefore, investigating fundamental polymer–solvent interactions opens new possibilities for designing materials with precisely tailored transport properties for next-generation applications.

Polymers can undergo a glass transition due to solvent exposure, where the chains shift from a supercooled state with nonequilibrium conformations^22–24^ into a system dominated by large-scale cooperative segmental motions induced by solvent interactions. However, compared to thermal annealing, the solvent-induced glass transition is more complex, requiring a change to the number of components in the system. Efforts to understand vapor sorption in glassy polymers are scarce despite its role in governing swelling, plasticization, and relaxation.^25^ Polymers in the glassy state undergo physical aging, in which their structure slowly relaxes toward equilibrium over time, affecting properties such as density, enthalpy, and mechanical response.^26–28^ Previous studies have shown that film thickness, surface-to-volume ratio, and interfacial interactions affect the thermal glass transition (*T*_g_) of polymer films. However, these effects have not been thoroughly investigated in solvent-induced glass transitions. The behavior of polymer–solvent swelling in thin films, particularly when exposed to solvent vapors for processing techniques like SVA,^29–31^ could similarly introduce significant complexities not observed in bulk materials.

Gas-penetrant sorption isotherms have been studied at high pressures, revealing a glass transition in polymers.^32,33^ Solvent sorption isotherms in bulk poly­(methyl methacrylate) in toluene vapor environments revealed that the glass transition solvent activity during absorption, *A*_g,Ab_ can be shifted by physical aging.^34^ Efremov and Nealey further demonstrated that the solvent ramp rate can shift the *A*_g,Ab_ in thin films using *in situ* ellipsometry.^35,36^ They systematically tracked residual solvent in a film after SVA and found that it correlated with the annealing history. However, the deviations observed in films annealed at activities well below *A*_g_ were attributed to residual gas sorption at the film surface rather than physical aging. While these earlier works present insightful results, the effects of molecular weight and solvent chemistry on the glassy behavior during SVA have not been investigated. Understanding SVA at low solvent activities could play a key role in the assembly of low χ BCPs,^37^ entrapped solvent-induced assembly,^38^ selective solvents,^39^ and the surge in application of sequential infiltration synthesis for BCP pattern transfer.^40–42^ More recently, we utilized the solvent absorption–desorption isotherms as a graphical method to determine kinetic pathways in the self-assembly of BCP thin films under SVA.^15^

In this paper, we aim to (i) conduct isothermal sorption measurements in thin films and (ii) investigate the effect of molecular weight and solvent parameters on the swelling behavior of glassy films. We demonstrate that the solvent vapor isotherms can be qualitatively linked to the Flory–Fox relation for the temperature-induced transition. The isotherms provide three descriptors (i) the glass transition activity, *A*_g_, (ii) the corresponding polymer fraction, ϕ_g_, and (iii) hysteresis, which scale with the glass transition temperature, *T*_g_. These quantities offer a unique, experimentally accessible view of the glass transition driven by solvent vapor activity. We utilized the excess osmotic pressure, ΔΠ, derived from hysteresis to capture physical aging in the glassy region. This adds a mechanical dimension to the thermodynamic picture and connects the swelling behavior to internal polymer stress states. In addition to the Flory–Huggins interaction parameter, all three parameters are dependent on the diffusivity of the polymer, while *A*_g_ was found to be especially sensitive to the vapor pressure of the solvent. Finally, we identify that physical aging indeed occurs in polymer thin films despite annealing at low activities. We classify four distinct isotherm types associated with different glassy states, offering a pathway toward predictive models that can more accurately account for plasticization, swelling hysteresis, and sorption dynamics in functional polymer thin films.

## Method

### Materials

Polystyrene (denoted PS), with molecular weights ranging from 1.2k to 750k Da (Table S1), and polystyrene-*block*-poly­(4-vinylpyridine) (denoted PS-*b*-P4VP, 94k-*b*-22k Da) were purchased from Polymer Source Inc., Canada. Amylene-stabilized chloroform was purchased from Sigma-Aldrich. Acetone and cyclohexane were purchased from Fisher Scientific. All chemicals were used as received. Silicon wafers with a thin native oxide layer of ∼2 nm were purchased from University Wafers. For measurements of 40 nm polystyrene films, silicon wafers with a native oxide layer of 300 nm were used.

### Sample Preparation

The polymers were weighed and dissolved in 5 mL of chloroform to prepare 1 wt % polymer solutions. The solution was then filtered through 0.45 μm syringe filters to remove dust or particulate matter. Thin films were prepared by spin coating an aliquot of the stock solution onto approximately 10 × 10 mm Si wafers at 2000 rpm for 45 s using a Laurell 650 M series spin-coater. Si wafers were plasma cleaned (atmospheric plasma) before use. As-cast thin film thicknesses were found to be ∼100 ± 5 nm. The thin films used to study the effect of thickness on glass transition ranged between ∼40 ± 5 nm and ∼300 ± 5 nm.

### Constructing Absorption–Desorption Isotherms

A custom-built solvent vapor annealing (SVA) assembly was used to construct the solvent vapor isotherms. Details of the SVA assembly are provided in the Supporting Information (Figure S1) and can be found in our previous report.^15^ Solvent activity, *A*, inside the chamber is defined as the ratio between the partial vapor pressure of the solvent vapor and the saturated vapor pressure of the solvent (*p*_*i*_/*p*_sat_). Saturation was ensured in the bubbler throughout the experiment (Figures S2 and S3). For the convenience of this study, the solvent activity is defined as

1 $$\mathrm{solvent activity}\left(A\right)=\frac{\mathrm{solvent vapor flow}}{\mathrm{total vapor flow}}$$

Thickness measurements were taken *in situ* using a Filmetrics F20 spectral reflectometer. First, we calibrated the thickness measurement system by establishing a baseline. Then, a thin film coated on a Si wafer is placed in the middle of the annealing chamber, convenient for taking accurate thickness measurements at the center of the thin film. Continuous thickness measurements started at 500 sccm N_2_ flow (*A* = 0). Then, the activity was increased by 0.025 increments and held at a particular activity for 30 s before the next increment. The thickness was recorded when the change was less than 2 nm at a given activity. Chloroform isotherms were collected up to *A* = 0.5. We observed dewetting of the film beyond this point; therefore, activities beyond this point are not considered. For cyclohexane and acetone, higher activities were chosen as the upper bound, as these solvents did not result in film dewetting. The desorption data were collected similarly with the activity reduced by 0.025 at each interval. The refractive index of the swollen films was accounted for when performing *in situ* thickness measurements. Once all of the data were collected, the thicknesses were converted to solvent fractions using the following equation and plotted against activity to construct the isotherms. The annealing chamber is housed on a thick slab of aluminum, which acts as a heat reservoir, maintaining the films at room temperature (∼18.5 °C). Temperature drift in the solvent bubbler due to endothermic cooling was measured *in situ*. In the low activity limit (<0.5), a total drift of ∼2 °C was observed during absorption, which amounted to negligible error in swelling. Near steady-state conditions (∼16.5 °C) were achieved at the onset of desorption. The details on endothermic cooling can be found in the SI (Figures S4 and S5).

2 $$\mathrm{solvent fraction}\left(⌀\right)=\frac{\mathrm{thickness at activity}x-\mathrm{initial thickness}}{\mathrm{thickness at activity}x}$$

## Results and Discussion

### 1 Relationship between Glass Transition Temperature (*T*_g_) and Glass Transition Activity (*A*_g_)

The polystyrene (PS)–chloroform system is selected to investigate the relationship between the molecular weight and the glass transition behavior of polymer thin films in solvent environments. Their χ parameters are known, and chloroform is considered to be a good solvent for PS. A continuous flow annealing system was incorporated for absorption–desorption isotherms constructions (Details in the Supporting Information, Figure S1). This system controls the partial pressure of the solvent and measures polymer swelling *in situ*.^15^ Glass transition studies were conducted using polymers with a wide range of molecular weights, with the average molecular weight (*M*_n_) spanning between 1.2 and 750 kg/mol. Figure 1(a) shows a typical isotherm for a PS thin film. Here, we continuously swelled the film by increasing the solvent vapor flow. The continuous flow through the solvent bubbler maintains a constant solvent partial pressure in the annealing chamber. We recorded the absorption and desorption thicknesses at a given activity and calculated the solvent fraction inside the film. The plot of activity (*A*) vs solvent fraction (φ) is the characteristic absorption–desorption isotherm of the polymer film.

**Figure 1:**
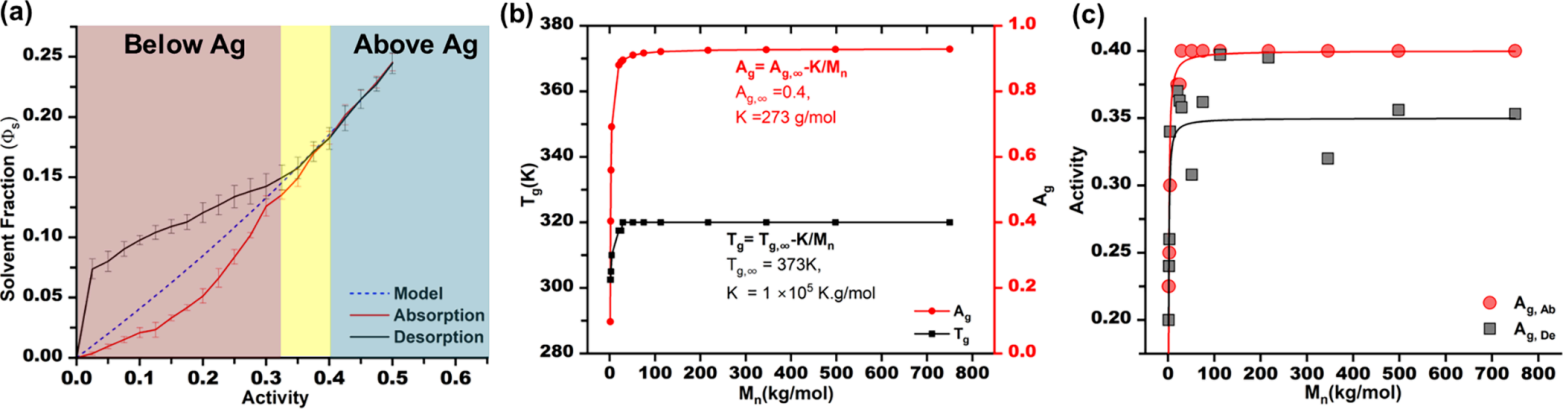
(a) Typical absorption–desorption isotherm (the blue dashed line represents the calculated Flory relationship for a film in equilibrium). (b) The relationship between the glass transition temperature, glass transition activity, and molecular weight of polystyrene. (c) The correlation between *A*_g,Ab_ vs *A*_g,De_.

At the point of spin coating, rapid solvent evaporation results in a glassy polymer film. During SVA, solvent molecules penetrate the polymer thin film, acting as plasticizers that enhance chain mobility, effectively reducing the glass transition temperature (*T*_g_) of the film. When the *T*_g_ approaches the level of the chamber temperature (*T*), the polymer film enters its solvent glass transition. Beyond this point, the polymer is completely plasticized into a viscoelastic state. At the onset of desorption, the solvent activity in the annealing chamber is reduced by increasing N_2_ while decreasing the solvent flow, which causes the polymer film to gradually deswell. The loss of plasticization reduces chain mobility and drives the polymer toward a kinetically frozen, glassy state, thereby increasing resistance to further solvent removal. This leads to an asymmetric desorption curve that deviates from the absorption curve, forming a hysteresis loop, as shown in Figure 1(a). The concave curvature is similarly observed in gas penetrant sorption of glassy polymers.^43^

We fit the viscoelastic region to the Flory eq (eq 3 ^44^), which describes the equilibrium polymer swelling at a given solvent activity, to reveal the glass transition and χ parameters.

3 $$\ln\left(A\right)=\ln\left(1-{⌀}_{p}\right)+\left(1-\frac{1}{N}\right){⌀}_{p}+\chi {{⌀}_{p}}^{2}$$

where *A* is the activity, ⌀_p_ is the polymer fraction at each activity, *N* is the degree of polymerization, and χ is the Flory–Huggins interaction parameter. This model is extended into the glassy region (below *A*_g_) to highlight their nonequilibrium behavior.

The solvent glass transition is taken as the point where the polymer swelling falls out of equilibrium. Hence, we identified two glass transitions, where the experimental swelling curve deviates from the Flory model: (i) desorption, *A*_g,de_ represents the lower bound, and (ii) absorption, *A*_g,Ab_ represents the upper bound. *A*_g,Ab_ coincides with the point of intersection between the absorption and desorption curves. This is consistent with the thermally determined glass transitions reported by Badrinarayanan et al.,^45^ where the point of divergence with the liquid equilibrium is lower when measured during cooling (desorption equivalent) compared to heating (absorption equivalent). Their observation was rationalized by a broader relaxation during cooling, which facilitates a shift in the onset of nonequilibrium behavior. In contrast, during absorption the film begins to swell from a glassy state with highly restricted solvent diffusion, causing nonhomogeneous plasticization, which could be the origin of the distorted nature of the curve and a delayed glass transition, *A*_g,Ab_. During desorption, the polymer is highly plasticized, leading to a more homogeneous distribution of solvent molecules, hence, a lower onset in nonequilibrium swelling, *A*_g,Ae_. Prior reports have indicated that *A*_g,Ab_ could be described as the softening point, *A*_s_, however, in this work we will adhere to the equilibrium and nonequilibrium definition, thus, coining this point as a glass transition.^34–36^

Our initial observation is that the glass transition activity^15^ closely follows the Flory–Fox approximation.^46^ The Flory–Fox eq (eq 4) is a derived relationship between the molecular weight of a polymer (*M*_n_) and its *T*_g_.^46^

4 $$T_{g}=T_{g,\infty }-\frac{K}{M_{n}}$$

where *T*_g_ is the glass transition temperature at a specific molecular weight, *T*_g,∞_ is the maximum glass transition temperature as the molecular weight approaches infinity, *K* is the Flory–Fox constant specific to the polymer system, and *M*_n_ is the number average molecular weight.

When the molecular weight (*M*_n_) of the polymer is low, chain-end dynamics dominate, leading to a higher free volume, greater segmental mobility, and a lower *T*_g_. The use of the Flory–Fox approximation for bulk polystyrene is reasonable, as the thin films used for this section were in the range of ∼100 nm. Consistent with our data, previous reports have shown that confinement effects become apparent when the film thickness is lower than 100 nm.^23,47,48^

We observed a similar trend in solvent-induced glass transition, *A*_g,Ab_ (Figure 1(b)), which correlates well with *T*_g_ (see SI, Figure S6 for more information). The anomaly at *T*_g_ ∼ 290 is likely due to thermal fluctuations (room temperature). We empirically determined an equivalent Flory–Fox parameter, *K* in terms of activity with a reasonably good fit (see SI, Figure S7 for more information). Interestingly, the lower *M*_n_ region shows an *A*_g_ and a hysteresis despite having *T*_g_ close to room temperature (Figure S8­(a),(b)). This baseline hysteresis could originate from resistance to swelling caused by thermal fluctuations. Yamamoto et al. developed a scaling description of swelling/deswelling in polymer networks in which the observed path dependence reflects a competition between the solvent osmotic driving force and internal elastic constraints including strand fluctuations and connectivity/entanglement constraints. In our thin films, when the nominal *M*_n_ is below the Flory–Fox threshold for a bulk glass at room temperature, chloroform plasticization raises the local *T*_g_ so that at the solvent fractions sampled the film exhibits a solvent-induced glassy/viscoelastic state. Under these conditions, analogous to network elasticity, entanglement constraints could contribute to a kinetic resistance to volumetric change.^49^

A significant observation from the upper and lower bounds transitions is that *A*_g,De_ fluctuates significantly, likely due to its dependence on effective χ. Figure 1(c) demonstrates that *A*_g,Ab_ and *A*_g,De_ in fact have a near-linear correlation when the swelling data are collected with isothermal increments. In previous studies, when these two limits were defined as *A*_s_ (the softening point) and *A*_g_ (glass transition), respectively,^34,36^ solvent activities were ramped at a constant rate, and the *A*_g,Ab_ is observed well above *A*_g,De_. As a result, *A*_g,Ab_ was largely seen as pathway-dependent, whereas *A*_g,De_ appeared as a more consistent transition. In contrast, the isothermal method applied here equilibrates swelling to the point of saturation at each activity. This effectively reduces the gap between the two, resulting in a stable *A*_g,Ab_. Thus, we infer that the isothermally determined *A*_g,Ab_—the point of intersection between absorption and desorption—provides a unified, robust, and practical swelling glass transition point.

The fluctuations in χ are verified in Figure 2(a). Analogous to the glass transition, the polymer films show a significant drop in χ and enter the negative regime at low *M*_n_. We attribute this to the increase in noncombinatorial entropy as chain length is reduced, which is embedded in χ (χ_eff_ to be precise), such as the rise in accessible chain conformations due to the lack of entanglements. In addition, the interaction parameters of the end-groups could be lower than those of the main chain repeat units. We observe an average χ of (0.05–0.12) in the plateau region beyond 100000 g/mol, which aligns with the reported value for the polystyrene-chloroform system, indicating a good solvent system where the coils are stretched.^50^ Below ∼25000 g/mol, the chain ends dominate the swelling behavior, which manifests as a plasticized film, as evidenced by decreased hysteresis and an increased polymer fraction at the glass transition, ϕ_g_ (Supporting Information, Figure S8). Here, ϕ_g_ rises sharply, indicating that fewer solvent molecules are needed to reach the viscoelastic state. Comparing the glass transition solvent/polymer fraction as a function of their thermal glass transition, *T*_g_ (Flory–Fox determined), we observe a linear correlation (Figure 2(b)). We find that the Chow model^51^ (see SI for more information), which describes the depression of *T*_g_ due to solvent infiltration, severely underpredicts the ϕ_g_, highlighting the complexity of the solvent-glass system compared to thermal analysis.

**Figure 2:**
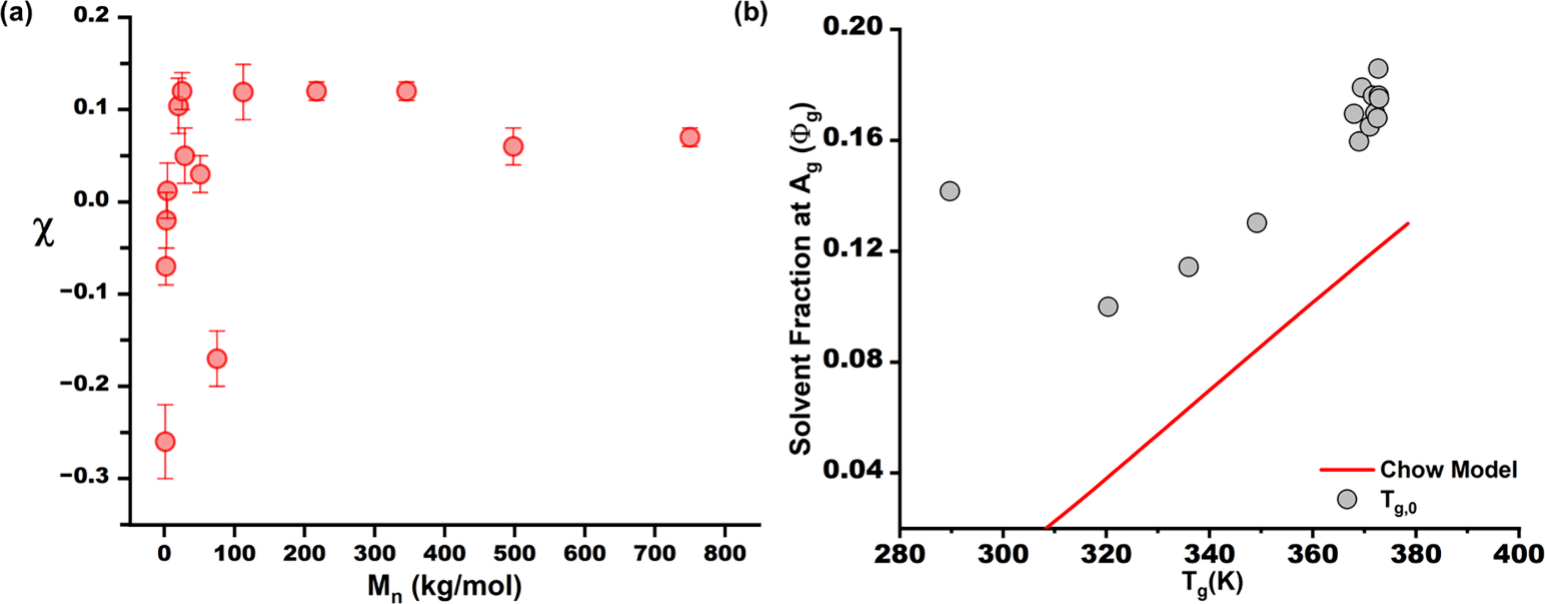
(a) Relationship of the Flory–Huggins interaction parameter and (b) solvent fraction at glass transition as a function of glass transition temperature, *T*_g_.

The asymmetry in absorption and desorption can be quantified using their equilibrium osmotic pressure (see SI, Figure S9 for more information). The expression *RT* ln *a* is the chemical potential, μ_1_ – μ_1_^0^, which when normalized by molar volume, *V*_m_ (molar volume of the solvent) yields the osmotic pressure, $\Pi =-\frac{\mu_{1}-\mu_{1}^{0}}{V_{m}}$ . Thus, eq 3 provides the equilibrium swelling (polymer fraction, ϕ_p_) for a given Π. In the glassy region (Figure 1a), nonequilibrium swelling (hysteresis) is observed, leading to an excess pressure, ΔΠ (eq 5) required for a given ϕ_s_.

5 $$\Delta \Pi =\left|\Pi_{\exp}-\Pi_{\mathrm{Flory}}\right|$$

The polymer in its glassy state requires excess osmotic pressure ΔΠ to initiate swelling (Figure 3a). However, in the absence of solvent (ϕ_s_ = 0), the osmotic pressure, Π_Flory_ is at its maximum, thus supplying sufficient driving force for swelling the glassy film. When ϕ_s_ > 0, Π_Flory_ rapidly falls leading to an increase in ΔΠ. By further increasing solvent activity, ΔΠ begins to fall, indicating that the thin film is creeping toward equilibrium. Furthermore, the onset of reduction in ΔΠ suggests that the film has softened due to plasticization (softening point) and finally undergoes a glass transition when ΔΠ = 0. Thus, ΔΠ quantifies the “glassy” behavior of the film and identifies softening trends. Thermal softening prior to *T*_g_ is analogously observed in glassy polymers.^34^ This observation validates that isothermal annealing well below the *A*_g_ of polymer thin films leads to severe physical aging.^34^ On the other hand, ramping solvent vapor pressure, as was done by Efremov and Nealey, leads to surface-limited sorption due to the slow kinetics in glassy films.^36^ Nonetheless, the implications of softening chains at low solvent fractions could open new avenues for solvent vapor annealing without approaching the order–disorder transition for block copolymers in the weak segregation limit.^52^

**Figure 3:**
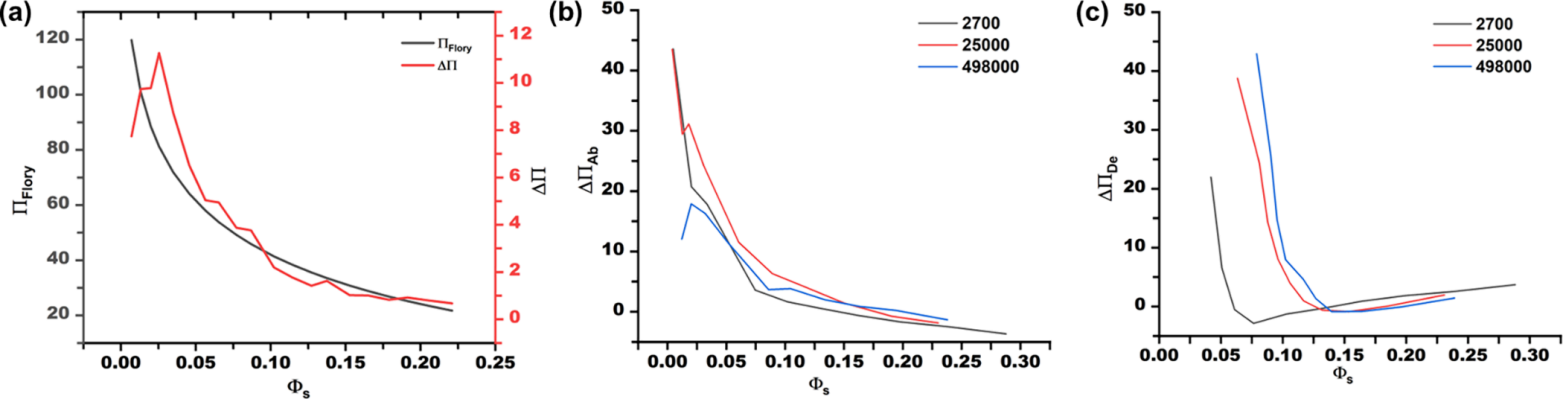
(a) Excess pressure as a function of solvent fraction compared to osmotic pressure obtained from the Flory relation (eq 3). Excess pressure at different regimes of molecular weight (g/mol) for (b) absorption and (c) desorption.

The ΔΠ of 2.7k PS in the desorption region is the lowest as expected due to the lack of entanglements. Interestingly, the absorption behavior in 2.7k PS appears to have a larger ΔΠ than 498k PS. We attribute this to the time that is taken to isothermally swell the glassier 498 K PS in absorption is significantly longer compared to the 2.7k PS, leading to severe physical aging, thus, bringing it closer to equilibrium. (see SI, Figure S9 for swelling curves demonstrating the kinetics of 100 nm ± 5 nm films at *a* = 0.7 for 180 s, across 2.7k, 25k, and 498k PS variants). During desorption, both films are in the viscoelastic state, which immediately falls out of equilibrium, as the films enter their glassy state. In addition, Yamamoto et al. showed, through scaling arguments, that shorter chains in networks experience a larger entropic penalty during swelling.^49^

To validate physical aging and diffusion of chains below *A*_g_ we tracked the morphology change of a polystyrene-*block*-poly­(4-vinylpyridine) (PS-*b*-P4VP) BCP before and after annealing at *A* = 0.3 (*A*_g_ ∼ 0.4). Here, it is clear that long-range order persists despite annealing below the glass transition.

As shown in Figure 4(a), the BCP is microphase separated into a spherical morphology after spin-coating with limited order (dispersed 2D FFT). Upon annealing at *a* = 0.3 for 1 h (Figure 4(b)), a modest improvement in morphology was obtained without undergoing glass transition (narrower 2D FFT). Here, the solvent evaporation rate determines the final morphology and properties of the material.^53,54^

**Figure 4:**
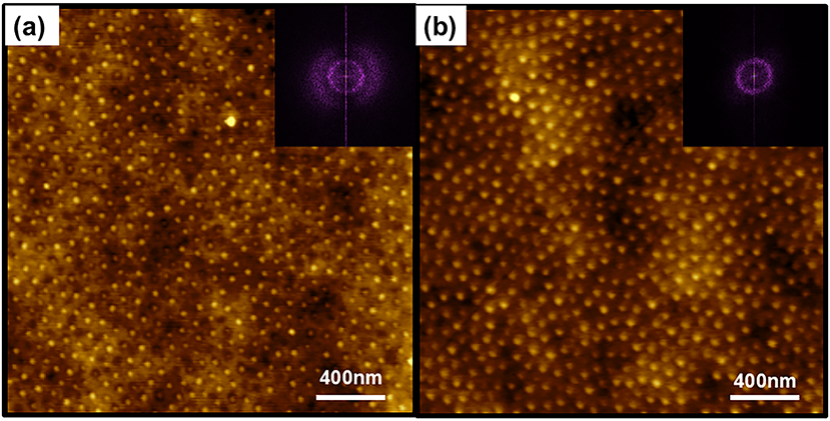
(a) PS-*b*-P4VP (94000-*b*-22000 g/mol) block copolymer thin film after spin coating and (b) after annealing at *a* = 0.3 for 1 h. Insets show the 2D Fast Fourier Transform of the AFM image.

### 2 The Effect of Solvent Interaction and Viscosity on *A*_g_, ϕ_g_, and Hysteresis

To investigate the effect of solvent parameters, we constructed isotherms for PS with a lower *M*_n_ (1900 g/mol) to ensure the films are easily plasticized, which is convenient for working with solvents with different interaction parameters against PS (Figure 5(a)). Chloroform is a good solvent for PS (χ ∼ 0.12) and expectedly exhibits a lower *A*_g,Ab_ and higher solvent fraction. On the other hand, acetone displayed a higher *A*_g,Ab_ and lower solvent fraction characteristic of a poor solvent (χ ∼ 0.6).^55^ Interestingly, cyclohexane demonstrated a dissimilar behavior despite having a large χ (1.16).^56^ In absorption, it demonstrates the poorest swelling below *A*_g,Ab_ as predicted. This is followed by a rapid increase in the solvent fraction above *A*_g,Ab_. This spike in solvent fraction is sustained during desorption as the film transitions back into a glassy phase. A similar scenario was reported in our previous work for the poly­(4-vinylpyridine)–chloroform system.^15^ The abrupt behavior in the isotherm arises from the kinetics of solvent penetration and can be rationalized through the relationship between diffusion and the glass transition. The diffusion of polymer chains is inversely related to *T*_g_ due to steric and secondary interactions that prevent segmental motion.^57^ Given that $D∼\frac{1}{T_{g}}$ where *D* is the diffusion constant and our observations that *T*_g_ ∼ *A*_g_ in Figure 1 and Figure 2, we extend this proportionality to the solvent glass transition parameters, $D∼\frac{1}{\left(A_{g},{⌀}_{g},\Delta_{\Pi }\right)}$ .

**Figure 5:**
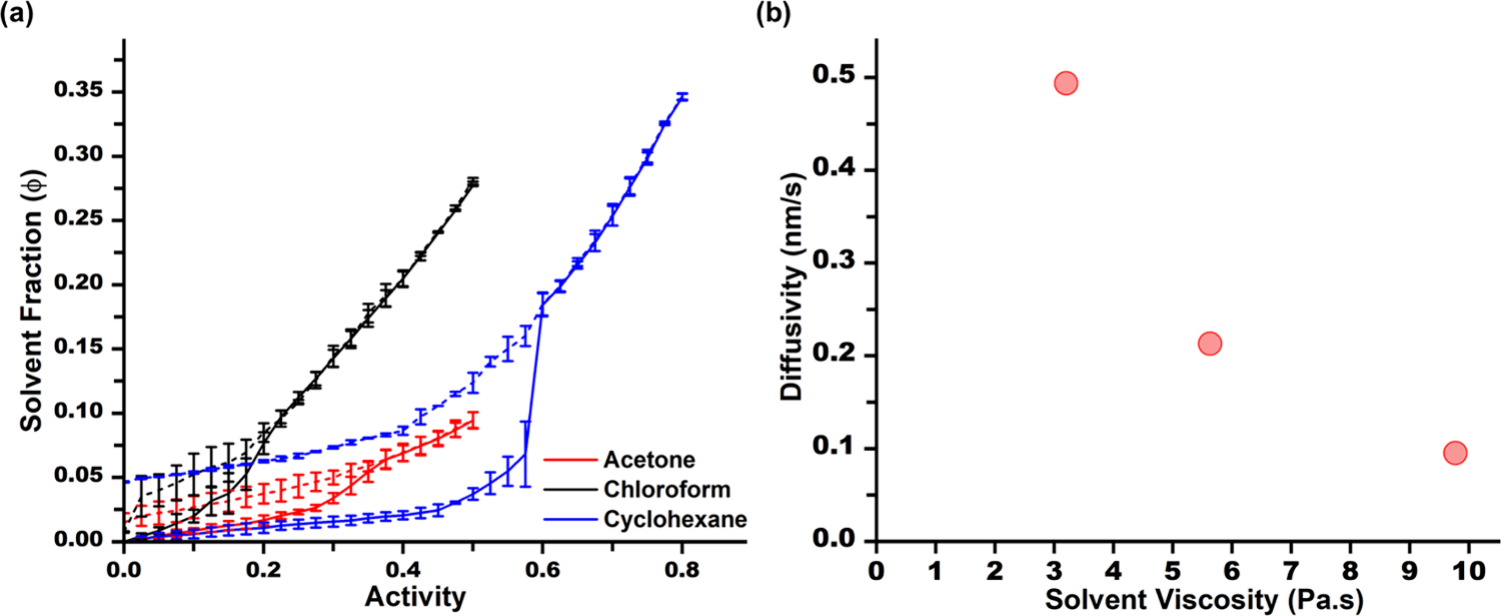
(a) Isotherms of PS with multiple solvents (the absorption is marked by the solid line whereas the desorption is marked by the dashed line) and (b) the solvent viscosity obtained from literature and the corresponding growth constant obtained from thickness measurements.

The diffusion constant of a polymer coil is given by the Einstein relation: $D=\frac{kT}{\zeta }$ where ζ is the friction coefficient, which scales as ζ ∼ *ηR*. Here, η is the viscosity of the medium, and *R* is the size of polymer coil. While limited diffusion of chains is observed under our experimental conditions (dewetting occurs only under prolonged solvent exposure), the viscosity of polymer solutions have been shown to scale with the solvent viscosity, η_*s*_.^58^ Hence, the isotherm parameters must scale with solvent viscosity, η_*s*_ ∼ *A*_*g*_, ⌀_g_, ΔΠ. Therefore, the limited diffusion of cyclohexane results in anomalously high *A*_g_, ⌀_g_, and ΔΠ tied to its higher viscosity (Supporting Information, Table SI2).

We further derived a diffusivity constant from our thickness measurements to verify the effect of viscosity (Figure 5(b)). We conducted an annealing experiment for each solvent at a fixed activity where the polystyrene film is above *A_g_* and results in approximately the same saturated solvent fraction, ϕ = 0.2 (chloroform, 0.4; acetone, 0.7; and cyclohexane, 0.65). The diffusivity constants were derived by fitting a Boltzmann function to the thickness data, which showed an inverse relationship to the available viscosity values, validating the effects of viscosity in solvent–polymer interactions.

## 3 Effect of Thickness on *A*_g_

It is well-established that the glass transition temperature (*T*_g_) of supported polymer thin films is depressed under nanoconfinement. This phenomenon, largely driven by a mobile surface layer, is notably independent of polymer molecular weight, suggesting it is a general finite-size effect rather than one dominated by chain-specific confinement.^47,48^ In thin films, the thermal glass transition temperature (*T*_g_) typically decreases once the thickness falls below a material-dependent characteristic length scale determined by substrate interactions and free-surface mobility.^59^ For strongly interacting substrates, this thickness limit can exceed 100 nm, while for polymers such as polystyrene that lack strong substrate attraction, the deviation from bulk *T*_g_ generally occurs below ∼100 nm.^48,60^ The current understanding emphasizes that confinement effects arise not as abrupt transitions but through a depth-dependent gradient in mobility, originating from the free surface and propagating tens of nanometers into the film.^23,36^ This gradient reflects cooperative segmental dynamics and gives rise to a continuum of confinement effects rather than a single crossover thickness.

To examine whether analogous behavior occurs in a solvent-swollen environment, we evaluated the thickness dependence of the solvent-induced glass transition activity (*A*_g_). We first performed thickness studies on 29000 g/mol PS, a molecular weight lying at the onset of *A*_g_ plateau behavior and consistent with the Flory–Fox limit for *T*_g_. In the initial data set, 70 nm films exhibited reduced *A*_g_ (∼0.35) and smaller hysteresis compared to the overlapping behavior of 100 and 130 nm films (*A*_g_ ≈ 0.4). Interestingly, solvent uptake above the glass transition was more pronounced in the 70 nm films, suggesting enhanced near-surface mobility under vapor sorption conditions.

We conducted another series at 20000 g/mol PS, expanding the range to 40, 100, 200, and 300 nm (Figure 6(a)). These data confirmed the earlier trend: the 40 nm film displayed distinctly lower *A*_g_ and reduced hysteresis, while the 100 and 200 nm films were nearly indistinguishable. The 300 nm film approached bulk-like behavior, exhibiting larger hysteresis and a higher degree of sorption above the glass transition. The fact that both molecular-weight series show the same systematic ordering—enhanced confinement at ≤70–40 nm, overlapping behavior at ∼100–200 nm, and bulk-like behavior by 300 nm—demonstrates that these differences reflect physical confinement effects rather than measurement variability.

**Figure 6:**
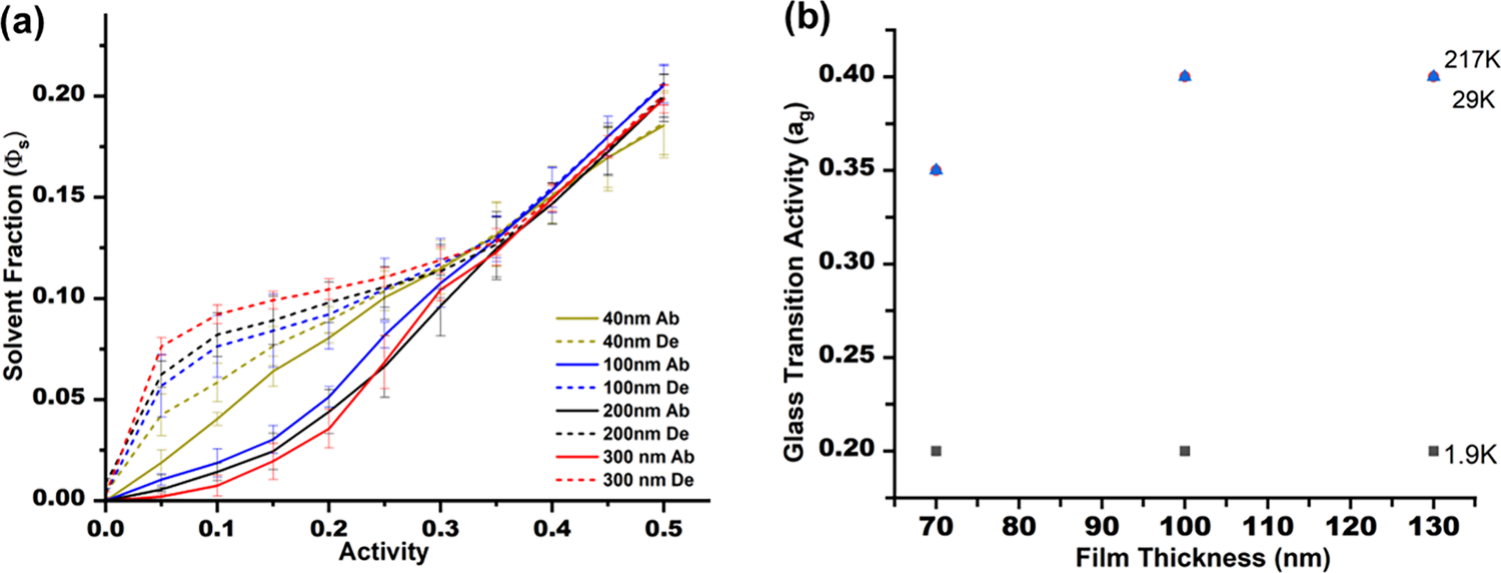
(a) Thickness dependent isotherms reflecting the effect on glass transition and hysteresis. (b) The glass transition variations based on thickness and molecular weight.

Importantly, these observations align with the established hierarchy of confinement length scales governing polymer thin films. Thermal *T*_g_ deviations typically emerge below ∼100 nm for PS.^48,60^ Recent work by Lan et al. has shown that probe-dependent translational diffusivity can be modified at thicknesses approaching ∼200 nm due to interfacial and dynamical gradients extending deeper into the film. Their study demonstrates that distinct dynamic observables “sense” confinement over different characteristic lengths, a framework that naturally explains why our solvent-induced *A*_g_ exhibits a confinement onset at intermediate thicknesses. *A*_g_ reflects cooperative segmental mobility under vapor-induced plasticization, which propagates from the free surface but does not extend as deeply as the length scale probed by molecular probe diffusion.^61^

Solvent primarily accelerates local segmental motions, effectively modifying their time scales and amplitudes, but the underlying mechanism of physical aging at low activity remains unchanged. Beyond 200 nm, the behavior becomes increasingly bulk-like. Finally, films of 1900 g/mol PS show negligible thickness dependence, consistent with the intrinsic molecular-weight limitation on *T*_g_ in oligomeric systems (Figure 6(b)). Overall, the results confirm that solvent-induced glass transition activity mirrors known confinement phenomena in polymer thin films and fits coherently within the established framework of thickness-dependent mobility gradients.

## 4 Summary, Limitations, and Future Directions

The findings of this study led us to four characteristic isotherms to consider in solvent–polymer interactions (Figure 7). We established that the shape of the isotherm is dependent not only on the solvent–polymer interactions but also on the diffusion kinetics of the solvent molecules. For a good solvent like chloroform (Figure 7a), we would expect an isotherm with a relatively low *A*_g_, and a smaller hysteresis. The solvent absorption will be higher in the viscoelastic region. The poor solvents, like acetone, will lead to low absorption and a higher glass transition due to weaker polymer–solvent interactions (Figure 7b). “Viscous” solvents such as cyclohexane result in a kinetically limited isotherm (Figure 7c), which shows a very low solvent absorption in the glassy region, followed by a strong spike close to the glass transition that continues into the viscoelastic region. The kinetically limited isotherm can also surface in strongly interacting polymers that have high *T*_g_. Finally, the plasticization or pre-existing plasticization in the films leads to an extremely low glass transition and smaller hysteresis (Figure 7d).

**Figure 7:**
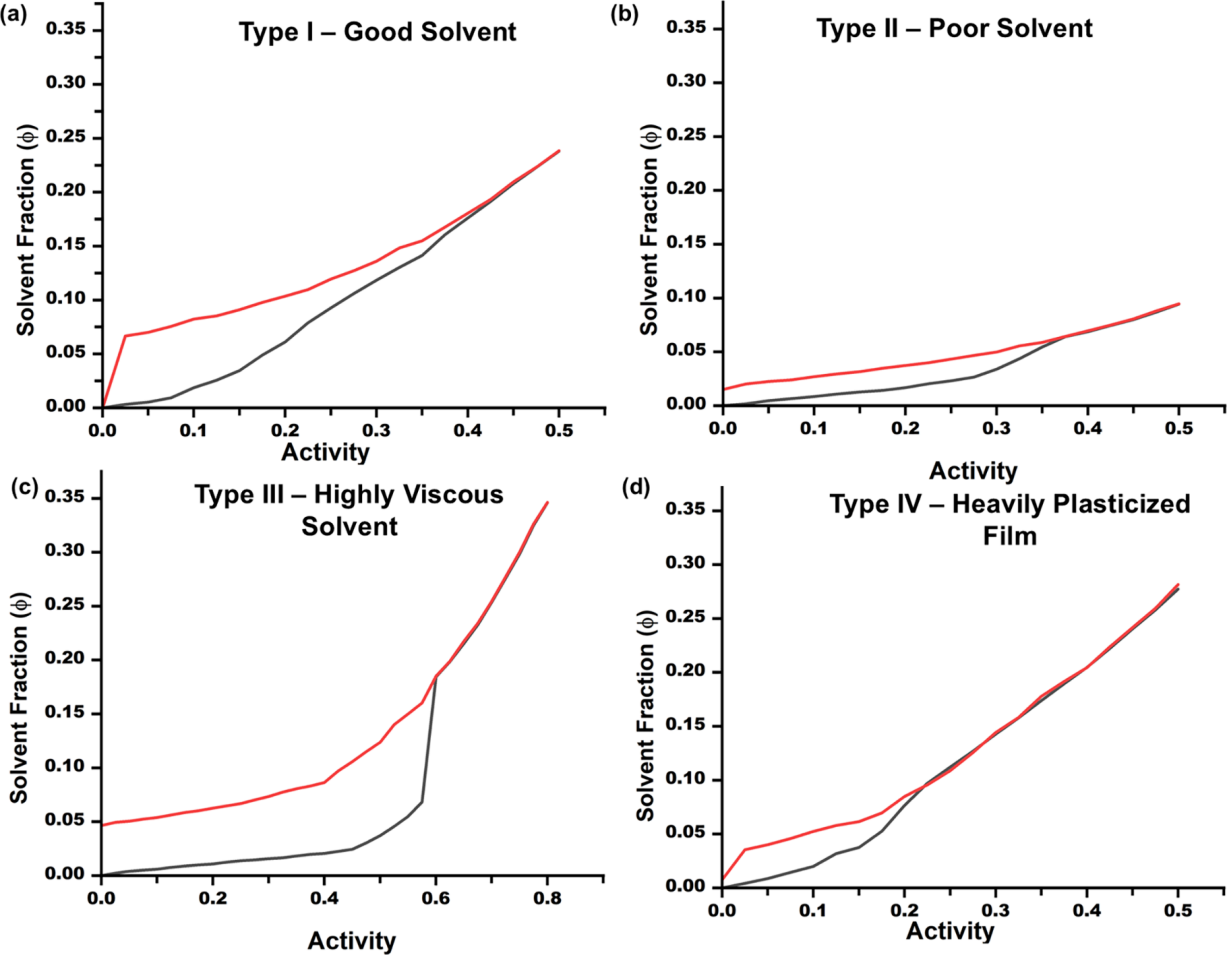
Types of isotherms. (a) Type 1: Good solvent (chloroform-PS); (b) Type 2: Poor solvent (acetone-PS); (c) Type 3: Poor diffusion (cyclohexane-PS or chloroform-P4VP); (d) Type 4: Highly plasticized (low *M*_n_ or supramolecular polymers). In all isotherms, the black curve marks the absorption regime while the red represents the desorption regime.

During these experiments, the swelling behavior of the polymer films can be transient due to multiple factors. Thus, the solvent activity ramp rate and physical aging will lead to differences in swelling. However, we have observed that the *A*_g,ab_ is robust when measured isothermally. Similarly, the overall behavior or shape of the isotherm remains consistent. Other factors such as humidity and substrate dewetting can play a role, especially when working with ultrathin films; thus, careful precautions in sample exposure time, endothermic cooling of the bubbler, inert gas purging, and film quality are critical. Constructing and identifying the type of solvent isotherm facilitate the deconvolution of polymer–solvent interactions, accounting for solvent/polymer kinetics in the film. This can be critical in developing a kinetic pathway for the SVA of BCPs and supramolecules. In this context, the isothermal behavior of BCPs and the graphical design of their annealing pathways for the targeted morphologies deserve further attention.

## Conclusion

This study demonstrates that absorption–desorption isotherms are effective in probing the solvent–polymer interactions in glassy thin films. A clear dependence on molecular weight was observed, consistent with the Flory–Fox relationship for the glass transition temperature. The isothermal nature of the solvent – polymer interactions leads to a more robust, uniform glass transition behavior in both absorption and desorption regions. Excess osmotic pressure analysis and BCP annealing at low activity highlight the potential for severe physical aging of thin films. This creates new opportunities for annealing low χ BCPs, selective solvent annealing, and performing sequential infiltration synthesis for BCP pattern transfer. Thin film effects on solvent-induced glass transitions correspond with thermal studies in which films below 100 nm begin to exhibit free-surface effects. Finally, we introduced four types of characteristic absorption–desorption isotherms that can be used as guidelines in determining the solvent–polymer interactions.

## Supplementary Material



## Notes

SVA schematic, solvent vapor saturation, glass transition curves, isotherms, and solvent parameters (PDF)
