# Supplementary material for: Understanding Solvent-Induced Glass Transition in Polymer Thin Films Using Absorption–Desorption Isotherms
Source: Macromolecules. 2026 Mar 2;59(6):3477–86. doi: 10.1021/acs.macromol.5c02242 (PMC13019668; doi:10.1021/acs.macromol.5c02242)
Supplement: Supplementary file 1 [file ma5c02242_si_001.pdf]

Supporting Information

Understanding Solvent – Induced Glass Transition in Polymer Thin Films Using Absorption – Desorption Isotherms

Nayanathara Hendeniya[1], Sharif Tasnim Mahmud[1], Shaghayegh Abtahi[1], Boyce S Chang[1,2*]

[1]Department of Materials Science and Engineering, Iowa State University, Ames, IA, United States.

[2]Micro-Electronics Research Center, Iowa State University, Ames, Iowa, USA

[*]Corresponding Author: boyce@iastate.edu

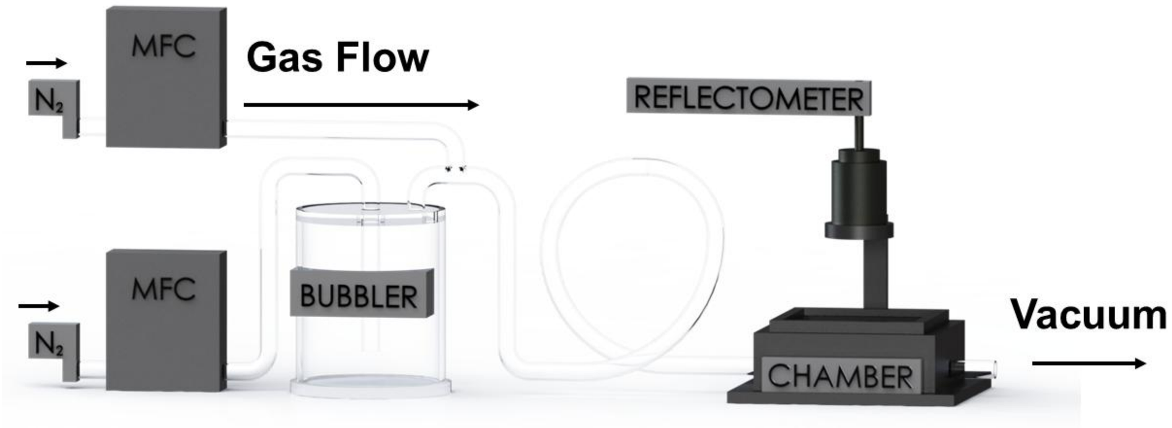

Figure S1: The Solvent Vapor Annealing set-up used for the experiments [1]

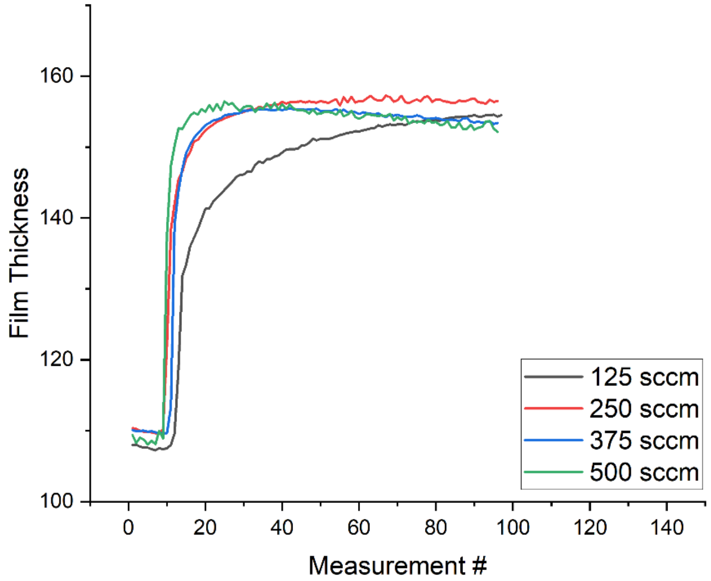

Figure S2: Swelling at different flow rates at a constant activity of 0.7. The flow rate was divided between the pure N2 and the solvent line to introduce solvent vapor to the chamber ensuring the same activity level. The drop in thickness observed in 375 and 500 sccm were due to film dewetting and potentially endothermic cooling of the bubbler considering a high activity was used.

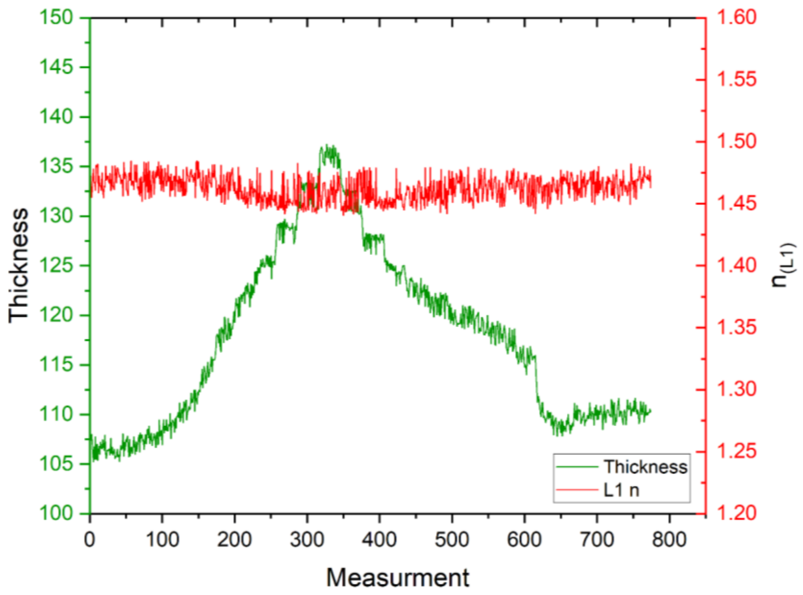

Figure S3: Thickness and refractive index of a polystyrene film recorded during the full isotherm sequence. The sequence is carried out in 0.05 activity increments, with a dwell time of 30 seconds at each activity.

Temperature Drift

Temperature change in the bubbler due to endothermic cooling was monitored in situ during an isotherm measurement (Figure S4). The cooling primarily occurred during the absorption phase (at the start of the experiment) and arrived near steady-state (~16.5°C) by the time desorption was initiated.

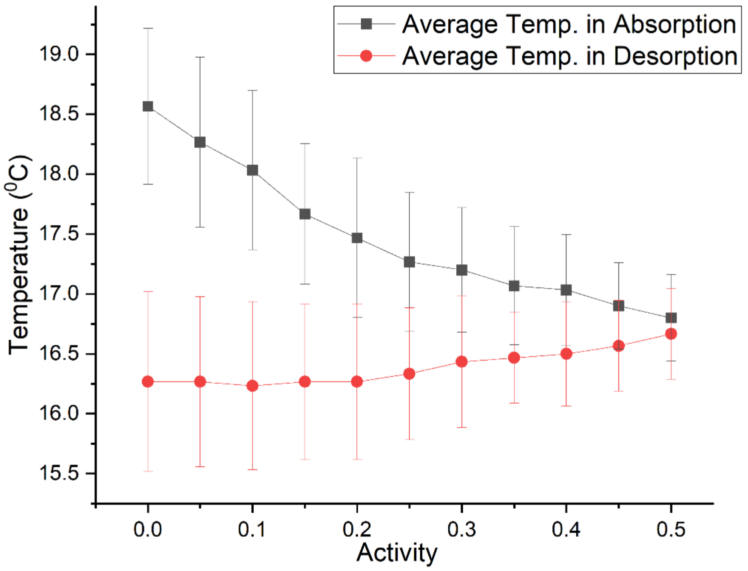

Figure S4: The Average temperature drift during an isotherm sequence.

The swelling change due to the 2°C temperature drift in the bubbler was calculated based on the change in vapor pressure of chloroform. The Antoine coefficients were used to calculate the vapor pressure of chloroform at 16.5°C, which resulted in 17.6kPa. Vapor pressure, P as a function of temperature is given by: $\log_{10} P = A - \left(\frac{B}{T+C}\right)$, where A= 4.207 bar, B=1233.129 bar, C=-40.953 bar, and T = temperature (K).[2]

This was used as the reference saturation pressure (steady-state) for calculating the drift in solvent activity during absorption. On average, the drift at each activity was <0.01. Finally, the activity drift (Δ activity) was converted into swelling by multiplying the corresponding drift with the derivative of the absorption curve :$\frac{d(solvent\ fraction)}{d(activity)}$. Here, the fractional thickness change due to activity drift was taken as the "temperature drift error", which was found to be negligible compared to the experimental thickness measurement error (Figure S5). Thus, the isotherms reported in this work will reflect steady-state conditions at 16.5°C.

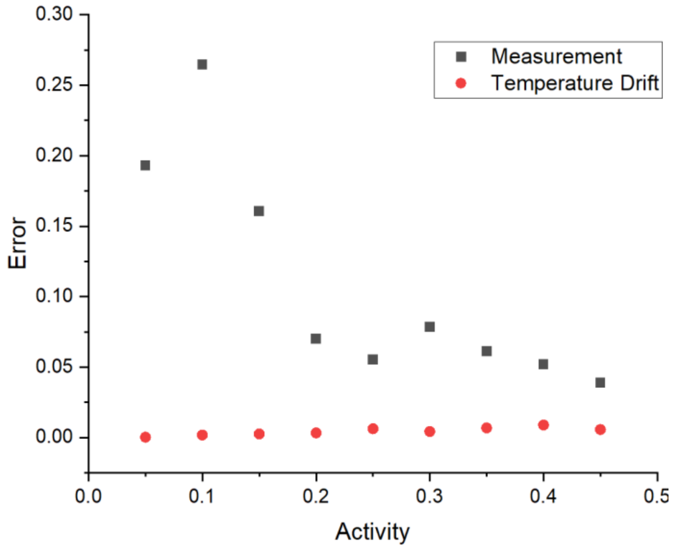

Figure S5: Comparing the relative thickness error during absorption from experimental measurement (black), and calculated temperature drift (red).

Chow Model

$$\ln\left(\frac{T_g}{T_{g,0}}\right) = [(1-\theta)\ln(1-\theta) + \theta\ln(\theta)]$$

$$\theta = \frac{V_p}{2V_d}\frac{\emptyset}{1-\emptyset}$$

$$\beta = \frac{zR}{M_p \Delta C_{pp}}$$

$T_g = glass\ transition\ temperature$

$T_{g,0} = glass\ transition\ temperature\ of\ the\ pure\ polymer$

$V_p = molar\ volume\ of\ the\ polymer$

$V_d = molar\ volume\ of\ the\ dilutant$

$\Delta C_{pp} = Excess\ transition\ isobaric\ specific\ heat\ of\ the\ polymer$

$M_p = Molecular\ weight\ of\ the\ monomer$

$z = Lattice\ coordination\ number$

$R = universal\ gas\ constant$

$\emptyset = volume\ fraction$

Heat transfer calculations

$\Delta H_{evaporation,\ chloroform}$ = 247 J/g, ~7.45g of chloroform consumed in a single experiment.

Total heat dissipated = 1840 J, in a span of 600 seconds. Therefore, the total heat transfer due to endothermic cooling, q_{endo} ~3W.

Conservatively, we assume only convective heating (ignoring conduction of the base with the Al slab) from i) natural convection of the aluminum thermal reservoir (h ~ 10W/m^2K), and ii) forced convection of the continuous gas flow (h ~ 20W/m^2K).[3] Convective heat transfer is given as q=hA($T_1 - T_2$) where A is the surface area of the slab (0.1 m^2) and h is the heat transfer coefficient. Temperature difference of 2 K can be estimated from the total heat (1840J) and specific heat capacity of a 1kg Al slab (~900 J/K). Taken together, $q_{convection}$ ~ 6W, which is a factor of two larger than cooling.

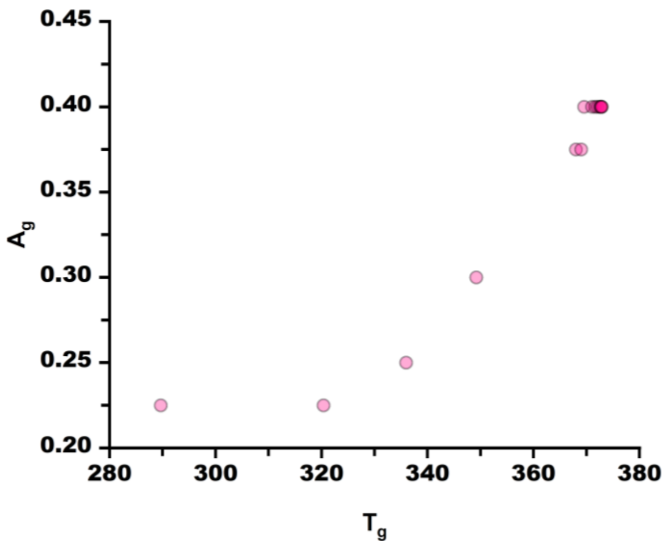

Figure S6: Ag and Tg correlation

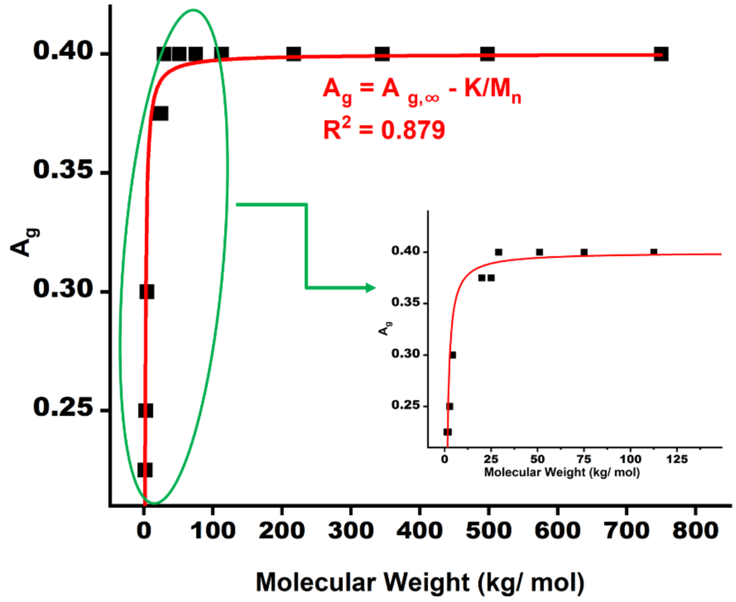

Figure S7: The function fit analogous to Flory-Fox equation using Ag values. The inset is an enlarged portion of the lower molecular weight region showing the transitions.

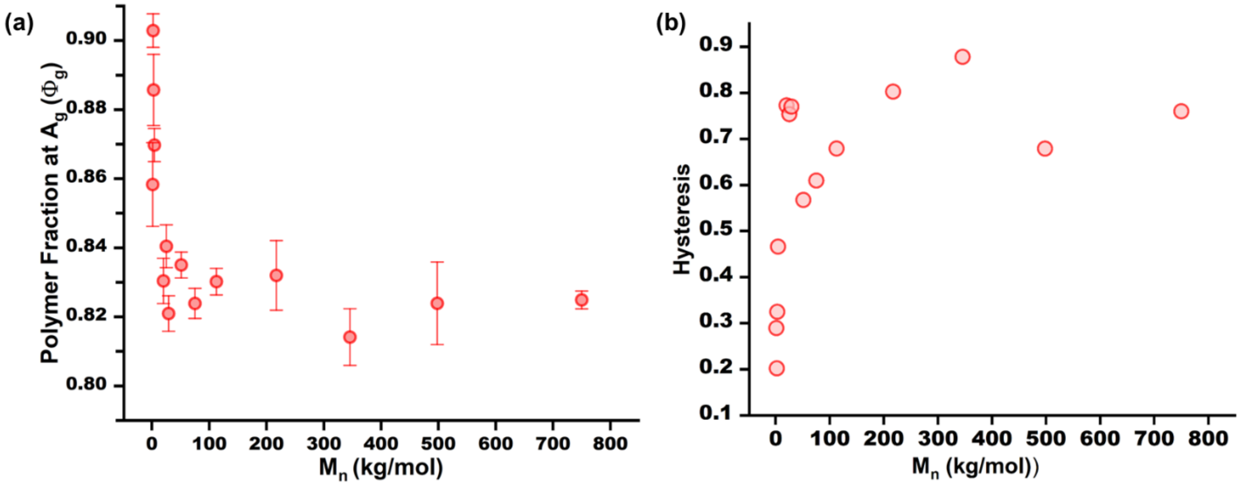

Figure S8: (a) Polymer fraction at glass transition. (b) Hysteresis of PS depending on the molecular weight. (The hysteresis calculation is described in detail in our previous work[1])

Table S1: Chain end chemistry and polydispersity table

Polymer Mn (g/mol)	*% Chain Ends*	*PDI*
1200	*17.36*	*1.12*
1900	*10.96*	*1.15*
2700	*7.71*	*1.16*
4200	*4.96*	*1.05*
20000	*1.04*	*1.13*
25000	*0.83*	*1.04*
29000	*0.78*	*1.1*
51000	*0.41*	*1.05*
75000	*0.28*	*1.05*
112500	*0.18*	*1.05*
217000	*0.09*	*1.04*
345500	*0.06*	*1.07*
498000	*0.04*	*1.08*
750000	*0.03*	*1.13*

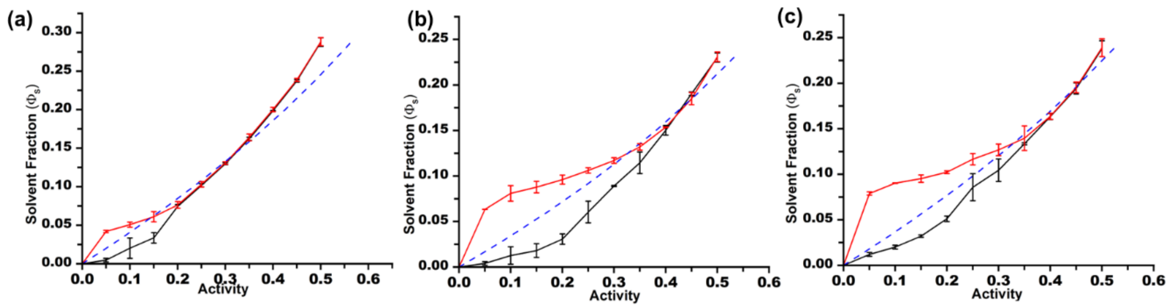

Figure S9: The behavior of isotherms and the Flory model in different PS films (a) 2700 g/mol, (b) 25000 g/mol (c) 498000 g/mol

Table S2: The key physical, chemical, and thermodynamic properties of the solvents used.

Type of Solvent	*Chloroform*	*Acetone*	*Cyclohexane*
Molecular Weight (Mw) g/mol	*119.37*	*58.08*	*84.16*
Melting Point (^{0}C) ¥	*-63*	*-94.8*	*7*
Boiling Point (^{0}C) ¥	*62*	*56*	*81*
Viscosity ξ (mP)	*5.63*	*3.2*	*9.77*
Density ¥ (g/cm^{-3})	*1.48*	*0.791*	*0.77*
Vapor Density §	*4.12*	*2*	*2.98*
Vapor Pressure ¥(mmHg)	*197*	*231*	*96.9*
χ_{PS}	*0.234*	*1.322*	*1.701*

¥ - Taken at 25^{0}C, ξ – viscosity given in millipoise. 1 Pascal seconds= 0.1 Poise, § - Vapor density is taken relative to air. Vapor density of air = 1

References

(1) Hendeniya, N.; Chittick, C.; Hillery, K.; Abtahi, S.; Mosher, C.; Chang, B. Revealing the Kinetic Phase Behavior of Block Copolymer Complexes Using Solvent Vapor Absorption-Desorption Isotherms. *ACS Applied Materials & Interfaces* **2024**, *16* (14), 18144–18153. DOI: 10.1021/acsami.4c00076.
(2) Singh, S.; Ghoshal, T.; Prochukhan, N.; Fernandez, A. A.; Vasquez, J. F. B.; Yadav, P.; Padmanabhan, S. C.; Morris, M. A. Morphology Engineering of the Asymmetric PS-b-P4VP Block Copolymer: From Porous to Nanodot Oxide Structures. *ACS Applied Polymer Materials* **2023**, *5* (11), 9612-9619. DOI: 10.1021/acsapm.3c02120.
(3) Ghahfarokhi, P. S.; Kallaste, A.; Belahcen, A.; Vaimann, T. Determination of Heat Transfer Coefficient for the Air Forced Cooling Over a Flat Side of Coil. *Electrical, Control and Communication Engineering* **2019**, *15* (1), 15-20. DOI: 10.2478/ecce-2019-0003.
